# FAK in Cancer: From Mechanisms to Therapeutic Strategies

**DOI:** 10.3390/ijms23031726

**Published:** 2022-02-02

**Authors:** Hsiang-Hao Chuang, Yen-Yi Zhen, Yu-Chen Tsai, Cheng-Hao Chuang, Michael Hsiao, Ming-Shyan Huang, Chih-Jen Yang

**Affiliations:** 1 Division of Pulmonary Critical Care Medicine, Department of Internal Medicine, Kaohsiung Medical University Hospital, Kaohsiung Medical University, Kaohsiung 80708, Taiwan; hsianghao.chuang@gmail.com (H.-H.C.); 1010362@kmuh.org.tw (Y.-C.T.); 1040239@kmuh.org.tw (C.-H.C.); 2 Division of Nephrology, Department of Internal Medicine, Kaohsiung Medical University Hospital, Kaohsiung Medical University, Kaohsiung 80708, Taiwan; a0928306234@gmail.com; 3 Genomics Research Center, Academia Sinica, Taipei 11529, Taiwan; mhsiao@gate.sinica.edu.tw; 4 Department of Internal Medicine, E-Da Cancer Hospital, School of Medicine, I-Shou University, Kaohsiung 82445, Taiwan; 5 Faculty of Post-Baccalaureate Medicine, College of Medicine, Kaohsiung Medical University, Kaohsiung 80708, Taiwan; 6 Cancer Center, Kaohsiung Medical University Hospital, Kaohsiung Medical University, Kaohsiung 80708, Taiwan

**Keywords:** focal adhesion kinase, metastasis, drug resistance, combination therapy, tumor microenvironment

## Abstract

Focal adhesion kinase (FAK), a non-receptor tyrosine kinase, is overexpressed and activated in many cancer types. FAK regulates diverse cellular processes, including growth factor signaling, cell cycle progression, cell survival, cell motility, angiogenesis, and the establishment of immunosuppressive tumor microenvironments through kinase-dependent and kinase-independent scaffolding functions in the cytoplasm and nucleus. Mounting evidence has indicated that targeting FAK, either alone or in combination with other agents, may represent a promising therapeutic strategy for various cancers. In this review, we summarize the mechanisms underlying FAK-mediated signaling networks during tumor development. We also summarize the recent progress of FAK-targeted small-molecule compounds for anticancer activity from preclinical and clinical evidence.

## 1. Introduction

Cancer is a leading cause of death, causing a substantial economic burden worldwide [1,2]. Despite advanced therapeutic strategies, prognosis remains unsatisfactory due to the rapid growth in cancer incidence and mortality across countries. Metastasis and tumor recurrence often result in cancer-related mortality, representing an enormous challenge that remains to be overcome.

**Figure 1:**
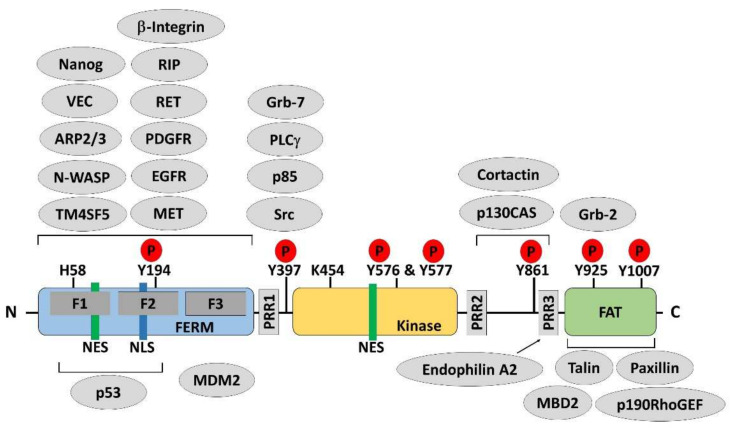
Protein structure and multiple binding partners of focal adhesion kinase. Focal adhesion kinase (FAK) is composed of an amino-terminal region containing a protein band 4.1–ezrin–radixin–moesin (FERM) domain, followed by a central kinase domain and a carboxy-terminal focal adhesion targeting (FAT) domain. Three proline-rich regions (PRRs) are embedded in the linker regions between these domains. Tyr397, Lys454, and His58 are important for FAK activation. Phosphorylation (P) occurs on several important tyrosine residues, as indicated, including the autophosphorylation site Tyr397, the Tyr576/577 residues in the activation loop of the kinase domain, and Tyr861, Tyr925, and Tyr1007 in the C-terminal domain. There are one nuclear export signal (NES) sequence and one nuclear localization signal (NLS) sequence in the FERM domain of FAK and one NES sequence in the kinase domain. Many proteins bind to FAK, regulating its functions or forming a complex, which is necessary for distinct biological processes. Phosphorylated Tyr397 is a well-known binding site for Src homology 2 (SH2) domain-containing proteins. The PRRs provide proline-rich sequences that bind with Src homology 3 (SH3) domain-containing proteins. The FAT domain is required to target FAK to the focal adhesion via binding to talin and paxillin.

Focal adhesion kinase (FAK) is a non-receptor tyrosine kinase that was identified about 30 years ago [3,4]. FAK is encoded by the protein tyrosine kinase 2 (*PTK2*) gene located on chromosomal region 8q24.3. The structure of FAK is composed of an amino-terminal band 4.1–ezrin–radixin–moesin (FERM) domain, followed by a kinase domain, and a carboxy-terminal focal adhesion targeting (FAT) domain. In addition, proline-rich regions (PRRs) are embedded in the linker regions between the FERM domain and the kinase domain and between the kinase domain and FAT domain (Figure 1). FAK was initially identified as a component of the integrin-mediated signaling axis [5,6], and growing evidence indicated that FAK is involved in the regulation of diverse cellular processes, including growth factor signaling, cell cycle progression, cell survival, cell motility, angiogenesis, and the establishment of an immunosuppressive tumor microenvironment (TME) through kinase-dependent and -independent scaffolding functions in both the cytoplasm and nucleus [7,8,9,10]. Due to increasing evidence supporting a role for FAK in malignant processes, FAK has been regarded as a promising pharmaceutical target for anticancer therapies.

The present study aims to summarize the various multiple roles played by FAK in tumorigenesis and to discuss the critical impacts of FAK signaling in cancer development and the survival of cancer associated cells. FAK is a promising drug target for cancer therapy, and the present study also summarizes the recent progress in the development of FAK-targeted small-molecule compounds and combination therapy strategies for cancer.

## 2. Functional Regulation of FAK

### 2.1. FAK Expression Regulation

Accumulating evidence indicates that FAK is often overexpressed in various cancers, and FAK levels have been correlated with poor prognosis in human cancers [8,10,11,12]. The expression of FAK is tightly controlled by multiple regulatory mechanisms. One FAK overexpression mechanism that has been identified in cancer is the copy number amplification of the 8q24.3 region containing the *PTK2* locus, which is adjacent to the *MYC* locus [13,14,15,16]. FAK expression is transcriptionally activated by nuclear factor-κB (NF-κB), which binds to the FAK promoter [17]. Nanog also binds the FAK promoter, which upregulates FAK expression [18]. It is also found that Nanog activity could be enhanced by FAK-mediated phosphorylation. This cross-regulation between FAK and Nanog might contribute to cancer malignancy [18]. Argonaute2 (AGO2) promotes FAK expression by binding to the FAK promoter in a micro-RNA biosynthesis-independent manner in hepatocellular carcinoma (HCC) [19]. Both *AGO2* and *PTK2* are located on chromosome 8q24.3, which is among the most frequently amplified regions in human cancers. The upregulation of AGO2 and the subsequent promotion of FAK expression may represent a positive-feedback loop resulting in high FAK expression in cancers. Furthermore, one of the Ets family proteins, polyomavirus enhancer activator 3 (PEA3), can upregulate FAK expression by binding to the FAK promoter [20]. However, p53 has been shown to suppress FAK expression [17,21,22]. Nuclear FAK promotes the proteasomal degradation of p53, mediated by the E3 ubiquitin ligase Mdm2, which might enhance cancer cell proliferation and survival [23].

In addition to transcriptional regulation, FAK expression is modulated at the post-transcriptional level through changes that affect FAK mRNA stability and protein translation. mRNA stability and translation are regulated by non-coding RNAs (ncRNAs), including transfer RNAs (tRNAs), ribosomal RNAs (rRNAs), small nuclear RNAs (snRNAs), piwi-associated RNAs (piRNAs), small-interfering RNAs (siRNAs), microRNAs (miRNAs), small nucleolar RNAs (snoRNAs), and long non-coding RNAs (lncRNAs) [24]. Several miRNAs, such as miR-7, miR-193b, miR-379-5p, miR-543, and miR-1298, have been shown to directly downregulate FAK expression and are regarded as prognostic biomarkers for human malignant diseases [25,26,27,28,29,30].

In addition to post-transcriptional regulation, FAK expression can be regulated by altering protein stability. FAK is degraded through proteasomal or protease-mediated pathways [31]. The E3 ubiquitin ligase Mitsugumin 53 (MG53) is responsible for the polyubiquitination of FAK, leading to FAK downregulation during myogenesis [32]. Moreover, FAK can be cleaved by several proteases. Tumor necrosis factor-related apoptosis-inducing ligand (TRAIL)-induced apoptosis triggers FAK cleavage by cysteine aspartate specific proteases (known as caspases), including caspases-3, 6, 7, and 8 [33]. c-Myc oncogene-induced cell detachment and cell death are also accompanied by the cleavage of FAK by caspases [34]. An aberrantly spliced FAK transcript resulting in the deletion of exon 26, removing the caspase cleavage site in the C-terminal domain, increases FAK protein stability, leading to sustained antiapoptotic signaling in breast cancer [35]. In addition, calpain, a calcium-dependent cysteine protease, has been shown to cleave both FAK and PYK2 [36,37]. The blockade of integrin signaling by degraded collagen fragments triggers the cleavage of FAK by calpain, resulting in focal adhesion disassembly in vascular smooth muscle cells [37].

### 2.2. FAK Activity Regulation

FAK is critically involved in the interaction between the cell and the extracellular matrix (ECM) [31,38]. As described above, FAK contains multiple functional domains that interact with other proteins to execute various biological processes (Figure 1). Integrin-mediated signaling induces FAK dimerization, leading to FAK autophosphorylation at Tyr397, generating an Src homology 2 (SH2) domain-binding site for Src-family kinases [39], and Tyr576 and Tyr577 in the FAK activation loop can be phosphorylated by Src [40,41]. In addition to Src, RET can phosphorylate the residues of Tyr576 and Tyr577 to activate FAK [42].

In addition to the kinase domain, the FERM domain serves as a major regulator of FAK activity through binding to the kinase domain, blocking the accessibility to Tyr397, and preventing autophosphorylation [41,43]. Experiments using fluorescent biosensors revealed conformational changes in the FERM domain upon ECM binding or interaction with phosphoinositide lipids, relieving autoinhibitory interactions [44,45]. Increasing the stiffness or tension associated with cell-ECM interactions through the strengthening of integrin signaling has also been shown to promote FAK activation [46,47,48], which is not only important for mechanotransduction but is also critical for tumor progression [38,46]. In addition to binding with ECM or phospholipids, the adhesion-dependent binding of the FERM domain of FAK with the transmembrane 4 L six family member 5 (TM4SF5) induces conformational changes in FAK, that relieve the intramolecular inhibition and promote FAK activation [49]. In addition to binding partners that accelerate the conformational changes in the FERM domain, growth factor receptors, such as MET, epidermal growth factor receptor (EGFR), and platelet-derived growth factor receptor (PDGFR), can also phosphorylate Tyr194 to relieve the autoinhibition and induce the FAK activation [50]. FAK Tyr925 phosphorylation mediated by G protein-coupled receptors (GPCRs) or receptor tyrosine kinases (RTKs) result in the association between FAK and the SH2 domain of growth factor receptor-bound protein 2 (Grb-2) in Rat 1a fibroblasts [51]. In addition, the adaptor protein Grb-2 mediates Src binding to FAK, facilitating FAK activation [52,53]. FAK activation also requires Janus kinase 2 (JAK2) activity following growth hormone stimulation [54]. Interestingly, increased intracellular pH-mediated His58 deprotonation also drives conformational changes in the FERM domain, allowing for access to Tyr397 and facilitating autophosphorylation [55].

Tyr397 phosphorylation is critical for FAK activation. SH2 domain-containing protein tyrosine phosphatase 2 (SHP2) is responsible for the dephosphorylation of pTyr397, inhibiting FAK activity [56]. In addition, the phosphorylation-dependent isomerization of protein tyrosine phosphatase (PTP)-PEST facilitates the interaction between PTP-PEST and FAK and the dephosphorylation at Tyr397 on FAK, leading to FAK inactivation [57]. However, SHP2 and PTP-PEST cooperate with Src to spatiotemporally control FAK activity, facilitating the dynamics of the focal adhesion complex, which promotes cell motility [56,58,59].

In summary, FAK activation is primarily controlled by the intramolecular inhibition of the FERM domain, FAK dimerization, FAK phosphorylation, or other mechanisms that remain to be deciphered.

### 2.3. FAK Nuclear Translocation Regulation

Subcellularly, the FAK is mainly distributed in cytoplasm and principally integrates and transduces signals from integrin or growth factor receptors to execute biological processes in cancer cells. Recently, nuclear FAK has received much attention, in particular, as a scaffold for transcriptional regulatory complexes for gene expression or for different E3 ligases-associated complexes for the turnover of transcription factors [23,60,61,62,63]. Mechanistically, FAK promotes GATA4 and IL-33 expression, leading to reduced inflammatory responses and immune escape [61,63]. FAK synergizes with the E3 ubiquitin ligase Mdm2 to promote p53 degradation through the ubiquitin–proteasome pathway, leading to cancer cell growth and proliferation [23]. Therefore, FAK attends nucleus where it regulates gene expression to promote tumorigenesis [60,64].

FAK consists of multiple domains rendering FAK interacting with more than 50 proteins and granting FAK to act as a kinase or molecular scaffolds [65]. In addition to the functional domains, one nuclear localization signal (NLS) sequence and two nuclear export signal (NES) ones in FAK were identified (Figure 1) [23,66]. The NLS and NES in the FAK function in FAK shuttling between the nucleus and the cytoplasm, when the FAK attends at FAs, likely a pseudo-immobile form of FAK anchors on the plasma membrane, and remains in the cytoplasm. Lim et al. reported that the level of free FAK is increased in the cytoplasm when cells detach from the matrix. The FAK releases from FA and can translocate to cell nucleus [23]. Additionally, the x-linked apoptotic protein inhibitor (XIAP) facilitates active FAK in the FAs under the laminar shear stress. The shear stress triggers the translocation of FAK into the nucleus while the depletion of XIAP reduces shear stress promotes the phosphorylation of Tyr-576 [67]. Lim et al. also reported that more FAKs are present in nucleus while cancer cell exposes to apoptotic inducer agent such as staurosporine [23]. Treatment with H_2_O_2_ promotes FAK nuclear localization and differentiation in myocytes [68]. Furthermore, the protein inhibitor of activated STAT1 (PIAS1)-mediated Lys152 sumoylation of FAK activates its autophosphorylation and nuclear localization [69]. These results indicate that stress signals and loss of focal adhesion targeting might force FAK translocated from the cytoplasm to the nucleus. FAK in nucleus ascribes to cellular response to stress for cell survival. However, the detail mechanisms concerning nuclear translocation of FAK still need to be investigated.

## 3. Roles of FAK in Cancer Progression

**Figure 2:**
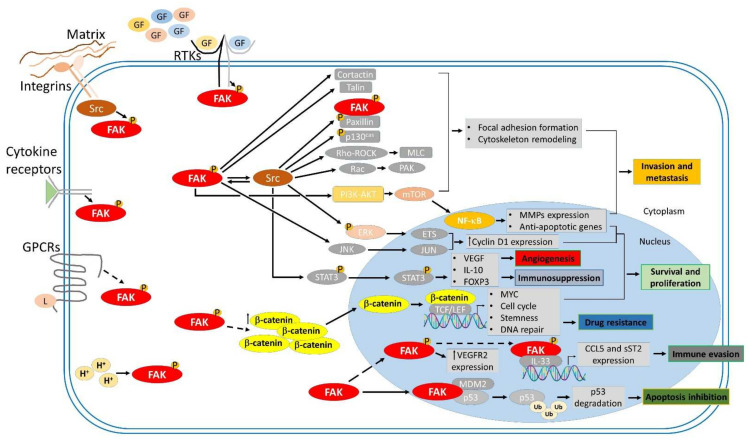
FAK-mediated signaling cascades involved in tumor progression. Focal adhesion kinase (FAK) is activated by Integrins, receptor tyrosine kinases (RTKs), cytokine receptors, G protein-coupled receptors (GPCRs), and changes in the intracellular pH (H^+^). FAK transmits upstream stimuli to downstream effectors through kinase-dependent and -independent signaling cascades, contributing to many biological processes involved in tumorigenesis, such as survival and proliferation, invasion and metastasis, angiogenesis, and immunosuppression.

A growing body of evidence found FAK overexpression in numerous human cancers correlated with poor prognosis. Recent studies have shown that FAK-associated integrin- and growth factor receptors mediated cell survival and cell motility through focal adhesion complex dynamics, which is kinase-dependent (Figure 1 and Figure 2). Additionally, FAK affects cancer cell survival and cancer stem cell proliferation through kinase-independent function of protein scaffolding [21,23,70]. The FAK promotes tumorigenesis not only through the maintenance of cell survival signaling and the enhancement of cell motility but also through other tumor-promoting processes. Accumulating evidence indicates that FAK acts as a critical central hub that fine-tunes diverse cellular processes, including growth, cell cycle progression, cell survival, cell motility, angiogenesis, the epithelial to mesenchymal transition (EMT), cancer stemness, and the establishment of an immunosuppressive TME (Figure 2) [7,8,9,10,64]. FAK is likely a strong contributor to the caner hallmarks summarized by Hanahan and Weinberg [71]. Signaling communications between tumors and the tumor-associated cells in the TME are critical for tumor progression [72]. In the TME, extracellular microenvironmental cues, which consist of growth factors, cytokines, and changes in pH, in addition to immobilized factors, such as integrins and changes in the ECM composition or stiffness, can activate enzymatic FAK to promote tumor growth and metastasis (Figure 2). In this section, we summarize the regulatory mechanisms through which FAK contributes to tumor progression.

### 3.1. FAK Promotes Cell Survival and Proliferation

FAK actioned to cell survival was first reported by Frisch et al. They found that the reinforcement of active FAK confer anoikis resistance in Madin Darbin canine kidney cells (MDCK) and immortalized human keratinocyte cells [73]. FAK is also involved in adhesion-mediated survival in cancer cells. The inhibition of FAK through the ectopic expression of antisense oligonucleotides or carboxyl-terminal domain of FAK (FAK-CD) results in the loss of adhesion and the promotion of anoikis in cancer cells, which is mediated by the Fas-associated death domain (FADD) and the caspase-8-mediated proapoptotic pathway [74,75]. FAK also cooperates with tumor necrosis factor (TNF) receptor-associated factor 2 (TRAF2), a really interesting new gene (RING) finger adaptor protein, to support cell survival and to bestow resistance to anoikis in breast cancer cells [76]. FAK binds to the death domain kinase receptor interacting protein RIP, by which the proapoptotic function of RIP is abolished [77]. Integrin-mediated FAK activation to endosomal signaling suppresses anoikis not only restricted in cell–ECM adhesions but also in endosomes with active integrins to confer anoikis resistance, anchorage independence, and metastasis [78]. FAK activates survival and proliferative signaling network mediated by the phosphatidylinositide 3-kinase (PI3K)-AKT-mammalian target of rapamycin (mTOR) axis to promote tumorigenesis, and FAK also activates NF-κB-mediated signaling to induce the expression of inhibitor-of-apoptosis proteins (IAPs) to sustain proliferation [79,80]. FAK is required for Ras- and PI3K-AKT-dependent survival and proliferative signaling during tumorigenesis [81]. Although p53 can downregulate FAK expression, interaction of FAK with p53 suppresses apoptotic cascade [82]. Beside cytoplasmic FAK, the FAK in cell nucleus forms a protein complex with Mdm2 and p53. Herein, the FAK-associated protein complex prompts the Mdm2-dependent ubiquitination and proteasomal degradation of p53 to transmit survival signaling [23].

In addition to survival signaling, FAK also promotes multiple cancer-driving pathways by augmenting proliferative activity. Integrin-mediated FAK activation leads to the upregulation of cyclin D1 through the activation of extracellular signal-regulated kinase (ERK), which drives the EtsB-mediated transcription upregulation of cyclin D1 [83]. These findings suggest that FAK activation results from integrin-mediated cell adhesion to the ECM. Subsequently, the integrin-based FAK activation promotes cell growth in both normal and cancer cells. FAK activation-mediated nuclear translocation of Yes-associated protein (YAP) is also characterized in insulin-like growth factor-1 (IGF-1), and its cognate receptor engaged proliferation in cancer cells [84,85,86]. FAK downregulated phosphorylation of c-Jun N-terminal kinase (JNK) also increases proliferation [87,88]. In addition to roles in cell survival and proliferation, FAK-mediated signaling also concerns with anti-senescence. By contrast, FAK inhibition leads to cellular senescence in cancer cells [81,89,90]. Targeting FAK in anticancer, it might take cell senescence by FAK inhibitors as a therapeutic strategy into account [91,92]. Thereafter, the combination treatment of FAK inhibitors with senolytic agents (drugs that selectively induce apoptosis in senescent cells) to selectively eliminate senescent cancer cells is a feasible therapeutic strategy.

### 3.2. FAK Enhances Cancer Cell Invasion and Metastasis

Cancer cell invasion and metastasis are key steps in the dissemination of cancer cells from a primary tumor to distant tissues or organs, which leads to progression and higher malignant grades [93]. This complex process involves the transition of tumor cells to a motile phenotype mediated by intracellular changes in focal adhesion dynamics and cytoskeleton rearrangement; the loss of E-cadherin during EMT to promote motile traits; the upregulation of matrix metalloproteinases (MMPs) to facilitate ECM invasion; and extracellular changes in ECM remodeling [93,94].

FAK acts as a central hub to fine-tune these cellular processes [31,95,96]. The principal mechanisms through which FAK governs cancer cell adhesion and migration through the formation of the focal adhesion complex and cytoskeleton remodeling (Figure 2) include: (i) functional interactions with Src family kinases [97,98,99,100,101]; (ii) the recruitment of talin to nascent focal adhesions complexes [102,103]; (iii) the formation of p130Cas/Cas/Crk complex [104,105,106]; (iv) functional interactions with small G proteins, such as Ras homolog family member A (RhoA), Rac family small GTPase 1 (Rac1), and cell division cycle 42 (cdc42), to regulate actin cytoskeleton reorganization [107,108,109,110,111,112]. Of actin remodeling, the FAK functionally interacts actin nucleation and elongation factors, such as cortactin, neural Wiskott–Aldrich syndrome protein (N-WASP), and the actin related protein 2/3 (Arp2/3) complex, respectively, with its PRR and FERM domain [113,114,115,116,117]. Besides actin remodeling, integrin signaling to FAK interactions with Grb-7 which is an adaptor protein comprising of an SH2 and pleckstrin homology (PH) domains is also an important regulatory mechanism underlying cancer cell migration [118]. Along with metastasis, the coordination of protein tyrosine phosphatase (PTP)-PEST and Src with FAK phosphorylation and dephosphorylation status manages focal adhesion turnover and modulates cell motility in both normal and cancer cells. Furthermore, the phosphorylation-dependent isomerization of PTP-PEST facilitates interactions of PTP-PEST and FAK and the dephosphorylation of Tyr397 on FAK for regulating cell migration, invasion, and metastasis [59].

Apart from the reorganization of the actin cytoskeleton, EMT programs regulate both morphological changes and molecular alterations in cancer cells, which results in the downregulation of epithelial markers expression and the increased expression of mesenchymal markers. The loss of E-cadherin as character of EMT is a critical step for the dissemination of cancer cells from a primary tumor [93,119,120]. FAK activation is the determinant step to progress EMT [26,121,122,123,124,125]. By FAK activation, the EMT transcription factors, such as Snail and Twist which induce EMT via the PI3K/AKT and MAPK signaling pathways, are expressed [126,127]. Twist and Zeb1 also promote integrin β1 expression to activate ITGB1-FAK signaling, suggesting the existence of a positive-feedback loop between ITGB1-FAK axis and EMT pathways [128,129]. FAK-Src activity influences the internalization of E-cadherin, reducing E-cadherin surface expression and disrupting cell-cell adhesion in tumor cells [130,131]. FAK also scaffolds endophilin A2 with its pro-878/881 motif. This suppresses endocytosis of membrane bound MMP MT1, leads to ECM degradation and promotes EMT [132,133]. In the context of involvement of FAK in EMT, miR-7 targets FAK expression and inhibits EMT and metastasis in breast cancer cells [26]. These findings indicate that FAK regulates both the transcriptional expression and cellular localization of E-cadherin to tune EMT and cell motility in tumor cells.

The regulation of MMPs on ECM remodeling is also regulatory mechanism acting in the invasion of motile cancer cells. Of FAK signaling to ECM degradation, the recruitment of the FAK-p130Cas-MT1 MMP (MMP-14) complex at focal adhesion sites results in MMPs remodeled ECM composition to alter invasive abilities of cancer cells [134]. FAK cooperates with Krüppel-like factor 8 (KLF8) to turn on MMP-14 and MMP-2 and indirectly regulates breast cancer cell invasion [135]. On the other hand, the interleukin-1-mediated activation of FAK and Src induce the MMP-9 expression promoting cell invasion in MCF-7 breast cancer cells [136]. Indeed, the FAK likely multifaceted regulator manages invasion and metastasis.

### 3.3. FAK Tunes Mechanotransduction

Mechanotransduction is a biological process in which eukaryotic cells can sense extracellular mechanical stimuli such as ECM stiffness, interstitial fluid pressure, and stretch and translate them into biochemical signals [137,138]. Integrins act as the major mechanosensors, which connect the intracellular cytoskeleton to the ECM, to link the extracellular mechanical cues to the cellular transcriptional machinery [139]. Integrin-FAK signaling recruits talin, vinculin, paxillin, and p130Cas as a functional complex-associated actin fibers at the focal adhesions (FAs). The FAs mediate the interaction of cell with ECM and are responsible for sensing the mechanical stimuli and translating them from the ECM to the cellular cytoskeleton [139]. It is also regarded as an outside-in signal transduction [139]. For mechanical forces, the constriction of actomyosin fibers induces conformational changes of FAK. In turn, FAK activates and converts mechanical stress to biochemical reaction inside of the cell [48]. In the FA, the cooperation of FAK with vinculin and paxillin modulates cytoskeletal contractility to transduce the force transmission from substrate stiffness [140]. Therefore, FAK acts as a homeostatic mechanosensor which spontaneously self-adjusts its activation status to match the ECM stiffness [141]. The ECM stiffness is a risk factor for tumor progression [46,142]. Mechanistically, increased matrix stiffness not only promotes integrin/FA activation but also elevates the level of β1 integrin through caveolin 1-atenuated endocytosis and subsequent lysosomal degradation of β1 integrin [143,144]. Caveolin 1 also acts as a mechanosensor in mechanotransduction, which is phosphorylated by FAK and Src and mediates membrane stability of lipid rafts, by which β1 integrins cluster and the FAs obtain maturation. This FAK-managed signaling promotes focal adhesion assembly, cytoskeleton organization, and YAP-mediated transcription [145,146,147]. Furthermore, individual FAs sense rigidity by applying fluctuating pulling forces to activate FAK-/phosphopaxillin-/vinculin-mediated signaling. Therefore, FAs act as mechanosensors to guide directed durotaxis, thereby increasing mechanotransduction [148]. Moreover, increased ECM stiffness drives β1 integrin-FAK signaling, which in turn, activates RhoA/ROCK1/MLC and RhoA/ROCK2/cofilin signaling cascades toward cancer cell motility [149]. YAP mediates mechanotransduction of extracellular stimuli to the transcriptional repertoire in nucleus [150]. In addition, YAP has been identified as an important downstream effector of FAK in mechanotransduction [147,151,152]. ECM stiffness promotes cells toward an EMT-like phenomenon with higher invasive and metastatic activity in cancer cells [153,154]. Furthermore, it has been characterized that increased collagen-matrix density promotes proliferation and invasion activity in nontransformed mammary epithelial cells through an FAK–Rho–ERK signaling network [155]. A similar phenomenon was also observed in glioma cells [156]. Noteworthily, FAK inhibition blockaded stiffness-induced phenotypic transformation and migratory activity of epithelial cells derived from murine MMTV-PyMT tumors. It indicated that integrin-FAK signaling upon ECM stiffness possesses the ability to drive EMT-related transformation and metastasis [157].

In addition to proliferation and cell motility, ECM stiffness-mediated FAK activation promotes the fibrotic and inflammatory events associated with pathological conditions [158,159]. The underlying mechanism showed that FAK acts through ERK to mechanically trigger the secretion of the monocyte chemoattractant protein-1 (MCP-1), a potent chemokine linked to human fibrotic disorders [158]. FAK inhibition alleviated MCP-1-mediated inflammatory cell recruitment, thereby reducing scar formation in vivo. Moreover, evidence shows that the tumor surrounding tissues are typically stiffer than healthy normal tissues [160] and increased matrix stiffness in the tumor microenvironment is correlated with poor prognosis [46,161]. FAK plays a critical role in mechanotransduction. Therefore, therapeutic targeting FAK activity may be a promising candidate for counteracting the deleterious effect of dysregulated mechanotransduction-related diseases.

### 3.4. FAK Drives Angiogenesis

Angiogenesis is the process of forming new blood vessels from the existing vasculature, which is essential for tumor survival and growth [162]. The endothelial cells (ECs) are a fundamental element for initiating angiogenesis. Global knockout of FAK or knock-in of kinase-dead ones results in the embryonic lethality with defects in blood vessel formation leading to hemorrhage and edema due to the blockage of EC proliferation, survival, and defects in EC polarity [163]. EC-specific knockout of FAK or EC-specific knock-in of kinase-dead ones also leads to embryonic lethality due to defects in blood vessel formation [164,165]. These indicated that endothelial FAK not only plays an important role in the regulation of chemosensitivity but also in angiogenesis. The mechanistic investigation showed that FAK control angiogenesis through catalytic activity-dependent and scaffolding-dependent manners. The scaffolding functions of FAK maintain the cell proliferation and kinase activity-dependent signaling cascades cell survival, cytoskeleton organization, and polarity in ECs [164,166]. FAK inhibition via protein downregulation or kinase inactivation decreases postnatal angiogenesis in adult mice [62]. On the other hand, the mechanistic investigation finds that nuclear FAK promotes vascular endothelial growth factor receptor 2 (VEGFR2) expression in a kinase activity-dependent manner, which is a regulatory mechanism underlying adult EC-mediated angiogenesis [62]. In the context of angiogenesis in tumors, FAK upregulates VEGFR2 expression in EC and promotes angiogenesis in triple-negative breast cancer [167]. Additionally, Krüppel-like factor 8 (KLF8) cooperates with FAK to promote VEGFA expression consequent to angiogenesis and tumor growth [168]. Moreover, lung EC-FAK stabilizes adherens junctions (AJs) to maintain lung vascular barrier function through regulating the balance between the activities of RhoA and Rac1 [169]. Therefore, FAK controls vascular morphogenesis and the regulation of EC survival and functions through the cooperation between catalytic activity-dependent and scaffolding-dependent manners. 

The FAK also acts a critical role in tumor angiogenesis for tumor survival and development [162,170]. Tavora et al. reported that the depletion of EC-FAK suppresses tumor growth and abolishes tumor angiogenesis by impairing VEGF-induced Akt phosphorylation and neovascularization in adult mice [170]. Besides the tumor cells, the tumor-associated ECs expressed more mRNA and higher protein levels of FAK as well as higher levels of FAK Tyr397 phosphorylation [171,172]. Failing in FAK phosphorylation at Tyr397 by the induction of *ECCre+;FAK^Y397F/Y397F^*-mutant mice reduced tumor growth and angiogenesis [173]. Mechanistically, the FAK-Y397F mutation blunts VEGFA and angiopoietin 2 (Ang2)-stimulated cell proliferation, survival, migration speed, and in vitro vessel sprouting of blood vessels. Therefore, targeting FAK might be a potent strategy turning down angiogenesis [174,175]. In addition, accumulating evidence has shown that the pharmacological inhibition of FAK prevents angiogenesis and suppresses tumor progression in animal models with human cancer cell implantation [172,176,177,178,179]. These results indicated that FAK plays a determinant role in angiogenesis for tumor growth. In addition to EC proliferation and migration, vascular permeability also is a critical driving force for angiogenesis [180]. The vascular permeability is controlled by the cell–cell adhesive junction formed by ECs. It was found that VEGF-induced vascular permeability is regulated by FAK [181]. The genetic or pharmacological inhibition of FAK in ECs blunted VEGF-stimulated microvascular hyperpermeability downstream of VEGFR or Src activation in vivo. Mechanistically, VEGF regulate vascular permeability by integrin-independent FAK activation. The FAK FERM domain interacts VE-cadherin, resulting in FAK attending at cell–cell junctions. In the cell–cell junctional complex, the FAK mediates phosphorylation of β-catenin. As a consequence, the dissociation of VE-cadherin–β-catenin and breakdown of EC AJs occur [181]. Apart from EC, the FAK in other cancer-associated stroma cells such as pericytes, platelets, and fibroblasts has angiogenic potential through distinct mechanisms. Kairbaan Hodivala-Dilke and her colleagues found pericyte FAK as a negative regulator of Gas6-Axl-AKT signaling for tumor angiogenesis and growth. FAK deletion in pericytes suppressed VEGF-, PDGF-B-, or PlGF-stimulating angiogenesis in vivo. However, the loss of FAK in pericytes results in pericytes hypersensitive to exogeneous Gas6 for Gas6-Axl-AKT-Cyr61 signaling to drive angiogenesis [182]. They also further characterized the significance of FAK Tyr861 phosphorylation in pericytes to tumor angiogenesis. Tumor growth and blood vessel density were reduced in *PdgfrβCre+;FAK^Y861F/Y861F^* but not *PdgfrβCre+;FAK^Y397F/Y397F^* mice. They also found that the altered secretome of *FAK^Y861F/Y861F^* pericytes increased necrosis of Lewis lung carcinoma (LLC) cells in early-stage tumors [183]. The same group also characterized the significance of FAK signaling in cancer-associated fibroblasts (CAFs) to angiogenesis and tumor growth. They reported that the depletion of CAF-FAK reduced the tumor blood vessel density in late-stage tumors. However, FAK-null CAFs changed the chemokine production, which triggered the metabolic reprogramming of cancer cells to support tumor growth [184]. In summary, FAK signaling acts as a crucial driver for angiogenesis and tumor growth. As a result, the therapeutic targeting FAK may be a promising strategy to suppress angiogenesis and a desirable therapy for cancer treatment. 

### 3.5. FAK Facilitates Immunosuppressive Tumor Microenvironment 

Tumor microenvironment (TME) is a complicated environment composed of heterogeneous stromal cells, including endothelial cells, fibroblasts, pericytes, and immune cells, and acellular components, including immobilized scaffolds such as ECM and soluble factors, including cytokines, chemokines, growth factors, and hormones, that surround tumors. TME has been known to play a crucial role in tumor initiation, progression, and metastasis [185,186]. Signaling communications between tumors and the tumor-associated cells in the surrounding TME are critical for these processes [72,187]. 

Mounting evidence has demonstrated the role of FAK in promoting TME remodeling for tumor progression and worse malignancy through enhanced propagation of cancer cells and cancer-associated stromal cells, expanding angiogenesis, increased ECM deposition (mechanotranduction), elevated cell motility for metastasis malignancy, and establishment of immunosuppressive TME. In tumor progression, heterogeneous immune cells play distinct roles in promoting either clearance or immune evasion [188]. Tumors or tumor-associated cells secrete various cytokines, chemokines, and ECM proteins to construct immunosuppressive environment and to disrupt tumor immune surveillance [189]. FAK activation links ECM deposition to the fibrotic and inflammatory events as pathological fibrogenesis in skin [158,159]. It hints that FAK promotes the secretion of the MCP-1 via ERK signaling to human fibrotic disorders [158]. FAK inhibition prevents TNF-α–induced inflammatory VCAM-1 expression through the association between nuclear FAK and GATA4 with the C terminus of Hsp70-interacting protein (CHIP) E3 ligase-mediated GATA4 degradation [190]. In addition, Jiang et al. found that higher FAK activity contributes to high levels of fibrosis and poor CD8+ cytotoxic T cell infiltration, leading to an immunosuppressive TME in a pancreatic ductal adenocarcinoma (PDAC) mouse model. They also found that targeting FAK renders the previously unresponsive *p48-Cre;LSL-Kras*^G12D^*;Trp53flox/+* (KPC) mouse model of human PDAC mouse model responsive to the immunotherapy agents [191]. Therefore, it indicates that targeting FAK could promote immune surveillance and render tumors responsive to immunotherapy by preventing the fibrotic and immunosuppressive TME. Nuclear FAK promotes the expression of IL-33, and the soluble, secreted form of the IL-33 receptor, called soluble ST2 (sST2) in murine squamous cell carcinoma (SCC) cells. Furthermore, the FAK-IL-33 complex interacts with chromatin modifiers and transcriptional regulators, such as TAF9, WDR82, and BRD4, to promote NF-κB-mediated expression of chemokines, including CCL5, leading to an immunosuppressive TME [63]. A recent study showed that FAK regulates the chromatin accessibility of c-Jun to the enhancer of IL-33 to drive its expression [61]. Summarily, it is a promising strategy by adopting the combination therapy with FAK inhibitors and immunotherapy to alter the T-cell population of the TME, in turn promoting tumor regression.

### 3.6. FAK Confers the Drug Resistance

In addition to roles in general cell survival and proliferation, FAK is thought to mediate resistance to various cancer therapies. One standard of care for high-grade serous ovarian carcinoma (HGSOC) patients is the application of chemotherapy consisting of carboplatin (a DNA damaging reagent) and paclitaxel (a microtubule stabilizer) following cytoreductive surgery to eliminate residual tumor cells [192,193]. However, the prognosis of HGSOC patients with the cancer cells bearing high FAK expression is poor due to drug resistance [194]. Advancing in the involvement of FAK in drug resistance, Kang et al. reported that FAK promotes AKT-mediated YB-1 phosphorylation and CD44 expression to induce paclitaxel resistance in ovarian cancer cells, since FAK inhibition renders ovarian cancer cells sensitive to paclitaxel treatment [195]. Furthermore, Diaz Osterman et al. found the elevation of FAK Tyr397 phosphorylation in non-necrotic residual tumor bispies from HGSOC patients who received preoperative (neoadjuvant) carboplatin and paclitaxel chemotherapy and in cisplatin-resistant ovarian cancer A2780 and OVCAR10 cells compared with lower FAK levels in the parental cells [194]. They also isolated an aggressive ovarian cancer cell line through in vivo selection for aggressive ID8 growth in C57BL/6 mice, which harbor spontaneous gains in *Kras*, *Myc*, and *FAK* genes (KMF cells). The KMF cells have phenotypic cisplatin resistance and cancer stem cell (CSC) traits [194]. By FAK activation, the reinforcement of β-catenin-mediated pluripotency, detoxifying enzyme expression (such as aldehyde dehydrogenase), and DNA repair gene expression arise in the KMF cells. In regard to FAK activation, the enhancement of stemness-associated pathways, EMT activity, and active DNA damage repair machinery result in cisplatin resistance and cell survival. Several lines of evidence concluded that cancer stemness and the EMT play critical roles in drug resistance [196,197]. It implies that FAK-mediated nuclear translocation of β-catenin contributes to the development of cancer stemness and EMT progression as consequence drug resistance concurrents [198,199,200,201,202].

The Ras–RAF–MEK signaling pathway is often aberrantly upregulated in various cancers, and Ras and RAF are frequently mutated genes, contributing to a worse prognosis [203,204]. Hirata et al. showed that treatment with the BRAF inhibitor PLX4720 cascades integrin β1-FAK-Src signaling, which reactivates ERK and mitogen-activated protein kinase (MAPK) signaling in *BRAF*-mutant melanomas, resulting in melanoma-associated fibroblast-mediated ECM remodeling [205]. FAK-mediated survival signaling confers melanoma resistance to BRAF inhibitors. The coinhibition of BRAF and FAK has been used to block ERK reactivation in *BRAF*-mutant melanomas [205]. A similar phenomenon was observed in *BRAF*-mutant colorectal cancer cells, in which FAK activation enhances Wnt/β-catenin pathway activation to promote BRAF inhibitor resistance. The combined inhibition of BRAF/Wnt pathways or of BRAF/FAK pathways resulted in strong anticancer effects in both a cell-based model and a mouse xenograft model [206]. In addition to RAF inhibitors (dabrafenib, GDC-0879, or vemurafenib), MEK inhibitors (trametinib) can also activate FAK within a couple hours. The dual inhibition of RAF-MEK signaling and FAK signaling might represents a promising therapeutic strategies for treating *BRAF*-mutant cancers. In addition to tumor cells, the surrounding endothelial cells can regulate chemosensitivity. Hodivala-Dilke and colleagues found that the tumor cells sensitize to DNA damaging therapy by specific targeting of endothelial FAK in vitro and in vivo [207]. They showed that FAK is required for DNA damage-induced NF-κB activation and cytokine production in endothelial cells. The active FAK enhances the chemoresistance of tumor cells against DNA damaging reagents both in vitro and in vivo, suggesting that the TME can affect the response to anticancer therapies and render cancers resistant to treatment [208].

In addition to chemotherapies, FAK also contributes to radioresistance in many cancer cells through activation of JNK signaling, Wnt-β-catenin signaling, and DNA damage response signaling [209,210,211,212]. Therefore, molecules that target FAK might be considered potential adjuvant agents increase the efficiency of standard chemotherapeutics and radiotherapeutics.

## 4. FAK-Targeted Therapies for Cancer Treatment

Cancer is a leading cause of death and a major public health problem worldwide, and the cancer incidence and cancer-related mortality continue to increase. In the past, several researchers attempted to treat cancer by developing highly targeted inhibitors against single pathways. However, tumorigenesis typically involves multiple concomitantly dysregulated pathways [71]. Therefore, new generations of anticancer drugs that aim to inhibit multiple cancer-driving pathways or the use of combination therapies are currently considered a more suitable strategy for cancer treatment. FAK is highly expressed in numerous cancers, including ovarian, breast, pancreatic, lung, melanoma, prostate, colorectal, glioblastoma, and esophageal cancers, and FAK regulates the diverse tumorigenic pathways, making it an attractive drug target for cancer therapy.

**Figure 3:**
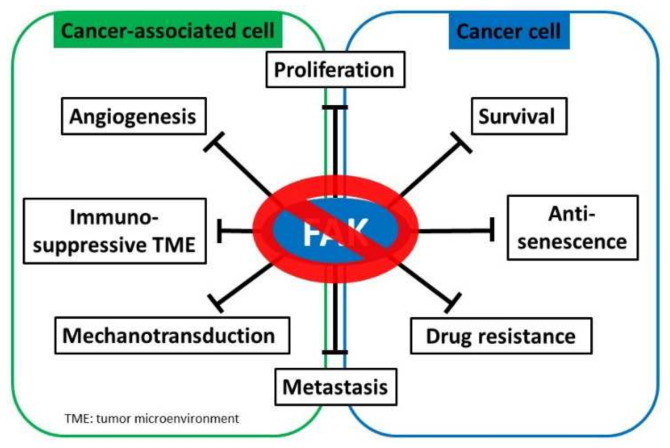
FAK inhibition serves as a potential cancer treatment strategy. Focal adhesion kinase (FAK) supports myriad oncogenic processes, and targeting FAK suppresses multiple critical biological capacities, such as survival and proliferation, drug resistance, metastasis, angiogenesis, mechanotransduction, and the establishment of an immunosuppressive tumor microenvironment to promote tumor progression.

Growing evidence indicates that targeting FAK is an effective approach to abolish cancer cell proliferation, metastasis, and immunosuppressive TME. FAK expression is regulated by transcriptional, post-transcriptional, and post-translational mechanisms to control physiological and pathological processes. FAK fine-tunes multiple signaling cascades through both kinase-dependent and kinase-independent scaffolding mechanisms to contribute to tumorigenesis (Figure 2). The role of FAK as a crucial hub for various tumorigenic signaling pathways, FAK inhibition has been a focus of much cancer research, and several FAK inhibitors have been discovered and synthesized. FAK inhibition is expected to suppress multiple biological capacities required for tumorigenesis (Figure 3). Most identified drugs target the catalytic kinase domain, although some also target the scaffolding activity. These potential FAK inhibitors, which are currently being tested in both preclinical studies and clinical studies, are listed in Table 1 and Table 2, respectively.

### 4.1. FAK Enzymatic Inhibitors

Most FAK inhibitors are small molecules targeting enzymatic or kinase-dependent fashion of FAK, such as ATP competitive kinase inhibitors targeting the ATP-binding site domain to block FAK kinase catalytic activity. These molecules are considered as KI type of inhibitors. The other type of inhibitors, blocking FAK kinase activity, are allosteric inhibitors, which target other sites of FAK and induce conformation change to block kinase activity. These molecules are regarded as aKI type of inhibitors.

BI-853520 (IN10018) is a highly selective and potent FAK inhibitor that blunts FAK autophosphorylation on Tyr397 with IC50 of 1 nM in PC3 prostate cancer cells and suppressed PC-3 prostate adenocarcinoma xenografts growth in nude mice [213]. Interrupting FAK signaling, treatment with BI 853520 dramatically reduced primary tumor growth and metastasis in various orthotopic breast cancer mouse models [214]. The compound is used in the clinical trial (phase I clinical trial #NCT01335269) to evaluate the safety and tolerability of BI 853520 monotherapy by defining the maximum tolerated dose (MTD) and recommending the dose for further trials in the development of this compound. This is a promising candidate for subsequent clinical trials.

GSK2256098 is a potent (Ki = 0.4 nM), selective, reversible, and ATP-competitive inhibitor of FAK to block enzymatic activity of FAK through targeting FAK Tyr397 phosphorylation. GSK2256098 treatment decreased cell viability, anchorage-independent growth, and motility of pancreatic ductal adenocarcinoma cells in a dose-dependent manner to suppress FAK-mediated AKT and ERK activation [215]. FAK-deficient platelets or treatment with FAK inhibitor, GSK2256098, prevented tumor rebound after cessation of antiangiogenic therapy [216]. It implies that FAK signaling in platelets is influenced. Herein, cell migration and angiogenesis contributing to the tumor microenvironment might be attenuated. Recently, safety, pharmacokinetics, and pharmacodynamics of GSK2256098 in healthy volunteers were investigated in a clinical trial (phase I clinical trial #NCT00996671). In addition, safety, tolerability, and MTD of GSK2256098 are estimated in the clinical trial (phase I clinical trial #NCT01138033).

NVP-TAC544 is a potent and ATP-competitive inhibitor of FAK to inhibit FAK activity. Treatment with the NVP-TAC544 blocked the bFGF-induced angiogenesis in WT but not i-EC-FAK-KO mice [217].

PF-431396 is an FAK/PYK2 dual inhibitor with an IC50 of 2 and 11 nM for FAK and PYK2, respectively [218]. Treatment with PF-431396 resulted in a dose-dependent inhibition of growth and anchorage-independent colony formation in both pancreatic cancer (PDAC) cell lines and mesothelioma (MPM) cell lines [219].

PF-573228 is an ATP analog, inhibiting FAK kinase activity with IC50 = 4.0 nM. This inhibitor is highly specific for FAK catalytic activity [220]. Treatment with PF-573228 suppresses cell growth, motility, and invasion of PDAC and MPM cell lines. In addition, PF-573228 treatment reduces the growth of tumor organoids from pancreatic cancer mice [219]. PF-573228 treatment suppresses cell proliferation and elicits senescence-like phenotype in lung cancer cell lines [89].

TAE226 is a potent ATP competitive inhibitor of several tyrosine protein kinases, in particular FAK and IGF-IR kinases with IC50 = 5.5 nM and IC50 = 120 nM, respectively [222]. TAE226 treatment suppresses the growth and invasion of glioma cells in vitro and prolongs the survival of nude mice with intracranial glioma xenograft through disrupting the dysregulation of AKT and MAPK signaling [222]. TAE226 treatment suppressed tumor cell growth and enhanced docetaxel-mediated cell cycle arrest in the taxane-sensitive and taxane-resistant cell lines. TAE226 administration significantly reduced tumor burden in vivo [234]. Therefore, TAE226 offers an attractive therapeutic approach for glioma and ovarian carcinoma.

The VS-4718 (PND-1186) is a selective, highly effective and reversible FAK inhibitor with IC50 of 1.5 nM in vitro and with IC50 of ~100 nM in breast carcinoma cells [223]. The low concentration of VS-4718 treatment did not affect c-Src or p130Cas tyrosine phosphorylation as well as cell proliferation in adherent cells but did block FAK and p130Cas tyrosine phosphorylation, promote caspase-3 activation, and trigger cell apoptosis in spheroids culture condition [223]. VS-4718 administration limited tumor progressions of the pancreatic ductal adenocarcinoma (PDAC) mice through reducing tumor fibrosis and decreasing numbers of tumor-infiltrating immunosuppressive cells. VS-4718 administration rendered the PDAC mice susceptible to T-cell immunotherapy and PD-1 antagonists [191]. VS-4718 are applied in clinical trials (phase I clinical trial #NCT01849744 and #NCT02215629) to evaluate its safety, pharmacokinetics, and the anticancer activity in nonhematologic and metastatic cancer patients and acute myeloid or B-cell acute lymphoblastic leukemia patients, respectively.

VS-6062 (PF-00562271) is an ATP-competitive, reversible, and FAK and Pyk2 dual inhibitor with IC50 = 1.5 nM and IC50 = 13 nM, respectively [224]. VS-6062 administration reduced FAK phosphorylation and tumor growth in vivo [224]. VS-6062 treatment inhibited the migration of tumor cells, CAFs, and CAMs in vitro. The administration of VS-6062 suppressed tumor growth, invasion, and metastases in PDAC mice model [225]. VS-6062 are tested in clinical trial (phase I clinical trial #NCT00666926) to determine its safety, pharmacokinetics, and pharmacodynamics of VS-6062 in patients with pancreatic, head and neck, and prostatic neoplasms. Those results showed that the MTD and RP2D of VS-6062 is 125 mg twice per day with food. VS-6062 exhibited time- and dose-dependent nonlinear PK and regarded as a potent inhibitor [226].

Defactinib (VS-6063) is an effective, ATP-competitive, reversible, and FAK and PYK2 dual inhibitor with IC50 = 0.6 nM for each kinase [235]. It is a very effective and specific inhibitor of FAK/PYK2. VS-6063 treatment reduced FAK Tyr397 phosphorylation in a time- and dose-dependent manner. The combination treatment with VS-6063 and paclitaxel significantly decreased proliferation and increased apoptosis, leading to reductions in tumor weight. Mechanistically, VS-6063 treatment reduced levels of AKT and YB-1 and enhanced chemosensitivity to taxane in taxane-resistant cell lines [195]. Defactinib (VS-6063) was tested in the clinical trial (phase I clinical trial #NCT00787033) to determine the safety, pharmacokinetics, and pharmacodynamics trial of VS-6063 in patients with advanced nonhematologic malignancies. The results were that VS-6063 has an acceptable safety profile. Treatment-related adverse events were mild to moderate and reversible [236]. It is a promising drug candidate. Another clinical trial (phase I clinical trial #NCT01943292) was set to determine the response of VS-6063 in Japanese patients with advanced solid tumors. The result is that VS-6063 was well tolerated at all dose levels investigated in this first-in-Asian study [235]. The drug was also used in phase II clinical trial (phase II clinical trial #NCT01951690) to determine whether VS-6063 (defactinib) treatment improves PFS within patients with KRAS mutant non-small cell lung cancer (NSCLC). The pharmacological effect of defactinib in the phase II trial (#NCT01951690) is monotherapy demonstrated modest clinical activity and failed to significantly improve outcome in heavily pretreated patients with KRAS mutant NSCLC [237]. Another phase II clinical trial (phase II clinical trial #NCT02004028) is to execute an open-label neoadjuvant (treatment with VS-6063 prior to mesothelioma surgery) study in subjects with malignant pleural mesothelioma who are eligible for surgery. The preoperative defactinib exposure was well tolerated and did not alter respectability or mortality compared to prior series.

1H-Pyrrolo(2,3-b) pyridine is an allosteric FAK kinase inhibitor by induction of DFG-loop conformation, binding to the hinge region of FAK and suppressing the kinase activity [227].

Compound 1 and 2 are non-ATP competitive, allosteric FAK inhibitors with IC50 = 0.96 μM [228]. The compounds used pyrazole ring interacts with the cationic side chain of Arg550 via cation–π interaction. One of the pyrazole nitrogens serves as a hydrogen bond donor to a water molecule. In addition, the polar chain is interacting with Gly563 or Asp564 to alter FAK structure, resulting in inhibiting FAK activity [228].

### 4.2. FAK Scaffold Inhibitors

Using ATP analogs to target the ATP binding pocket of kinase is a direct way to inhibit kinase activity. However, kinases share conserved sequences in their ATP binding sites such FAK and other tyrosine kinases, and the probability of off-target might be inevitable. Therefore, one way to avoid this problem is to directly interfere with FAK autophosphorylation. It specifically inhibits FAK activity but not other tyrosine kinases. Furthermore, FAK directly interacts with a number of critical proteins to transmit signaling for tumor cell survival. Therefore, targeting the key protein–protein interface of FAK with small molecules might be a feasible strategy to inhibit tumor growth. Summarily, these molecules are regarded as FAK scaffold inhibitors. 

1-(2-Hydroxyethyl)-3,5,7-triaza-1-azaniatricyclo[3.3.1.13,7] decane bromide (Y11) is a small-molecule inhibitor targeting Y397 site of FAK with IC50 = 50 nM. Y11 treatment effectively inhibits FAK autophosphorylation activity, reduces cell viability and the clonogenic activity of cancer cells, and suppresses tumor growth in vivo [231].

1,2,4,5-Benzenetetraamine tetrahydrocloride (Y15) is a small-molecule inhibitor targeting Y397 site of FAK with IC50 = 1 μM. Y15 specifically inhibits FAK Tyr397 phosphorylation and cell adhesion in a dose- and time-dependent manner. Y15 treatment effectively reduced breast tumor growth in vivo [232]. In addition, Y15 treatment reduced cell viability and clonogenicity of lung cancer cells. The administration of Y15 inhibited tumor growth of both RAS-mutant and EGFR mutant NSCLC. Mechanistically, JNK activation is a mechanism underlying Y15-mediated JNK activation to downregulate Bcl-2 and Mcl-1 [233].

Chloropyramine hydrochloride (C4) is a small-molecule inhibitor interrupting the protein-protein interaction of FAK with VEGFR-3. The IC50 value of C4 in various cancer cell lines ranged from 1 to 20 μΜ. C4 treatment inhibited VEGFR-3 and FAK signaling and abolished proliferation of a diverse set of cancer cell types in vitro. Furthermore, the administration of C4 reduced tumor growth in vivo [229].

Roslin 2 (R2) is an identified compound targeting the protein–protein interface between FAK andp53. R2 treatment decreased cancer cell viability and clonogenicity in a p53-dependent manner. R2 compound treatment upregulated p53 and its target genes, such as p21, Mdm-2, and Bax. Furthermore, R2 administration significantly inhibited tumor growth, disrupted formation of FAK and p53 protein complex, and upregulated p21 in vivo in a p53-dependent manner [230].

LD2-LD3-LD4 is a polypeptide capable of disrupting interactions between the paxillin LD motifs and the FAK FAT domain by competing interactions with FAK. The overexpression of LD2-LD3-LD4 prevents FAK localization at FAs, failing to transmit the integrin-mediated signaling. The overexpression of LD2-LD3-LD4 dramatically reduces FA turnover by absence of FAK in FAs and inhibits tumor cell migration and invasion [238].

### 4.3. FAK Inhibitors in Combination Regimes

Mounting evidence has shown that FAK acts the central hub to fine-tune diverse cellular processes, contributing to cancer progression. The outcomes of FAK inhibitors in clinical studies were not satisfied due to limited efficacy. Still, cancer is a difficult disease to treat, due to the genetic, cellular, and stromal complexity. Frequently, monotherapies targeting individual signal molecule or pathway failed. This may be a result of the presence of inherent or adaptive or acquired drug resistance. The roles of FAK in tumor progression not only involved in proliferation and survival but also contributed to drug resistance including chemotherapy, radiotherapy, or targeted therapy. 

As we mentioned above, FAK confers the drug resistance such as a chemotherapy composed of carboplatin and paclitaxel. Targeting FAK, cancer cells are substitutable to chemotherapy. Therefore, researchers investigated the clinical studies (phase I clinical trial #NCT01778803) to determine the efficacy of in combination therapy with defactinib (VS-6063) and paclitaxel in patients with advanced ovarian cancer. Defactinib is currently in ROCKIF Trial: Re-sensitization of Carboplatin-resistant Ovarian Cancer (phase I/II clinical trial #NCT03287271) to investigate the combination therapy with VS-6063, carboplatin, and paclitaxel in the treatment of patients with ovarian cancer. VS-4718 is adopted in clinical trial (phase I clinical trial #NCT02651727) to evaluate increasing the dose levels of VS-4718 administered in combination with gemcitabine and nab-paclitaxel in subjects with advanced cancer and to determine a recommended Phase 2 dose (RP2D) for further development of this combination in subjects with untreated advanced pancreatic cancer.

In addition to chemotherapy, targeted therapies against the Ras–RAF–MEK signaling pathway are simultaneously used to activate FAK signaling, which might reactivate ERK and MAPK signaling in BRAF-mutant cancer. Therefore, the dual inhibition of BRAF-MEK and FAK is able to effectively block ERK reactivation in BRAF-mutant cancer [205]. The series of clinical trials are set up to investigate the efficacy of combination therapy with BRAF/MEK inhibitor and FAK inhibitor. The clinical trial (phase I clinical trial #NCT03875820) about the combination of the FAK inhibitor, VS-6063, and the dual RAF/MEK inhibitor, RO5126766 in patients with advanced solid tumors aims to determine the MTD and RP2D. In addition, defactinib and VS-6766, a dual RAF/MEK inhibitor, are used in several clinical trials to determine the efficacy of combination therapy. The combination therapy is currently in the trial (phase II clinical trial #NCT04625270) to evaluate the effect of combination therapy of VS-6766 and Defactinib in recurrent low-grade serous ovarian cancer with and without a KRAS Mutation. The trials (phase II clinical trial #NCT04720417 and NCT04620330) about defactinib and VS-6766 for the treatment of patients with metastatic uveal melanoma and patients recurrent KRAS-mutant non-small cell lung cancer are still ongoing. The combination therapy with GSK2256098 and trametinib is currently in the trial (phase I clinical trial #NCT01938443) to assess the safety of combination treatment of GSK2256098 and trametinib in mesothelioma subjects [239].

The FAK also acts in immunosuppressive TME. Targeting FAK, cancer cells are susceptible to checkpoint immunotherapy [191]. This result hints that the FAK inhibitor in combination with immunotherapy might be substantiated in clinical trial drug regimen. The clinical trial (phase I clinical trial #NCT02943317) is applied to evaluate the safety, efficacy, PK, and PD of defactinib in combination with avelumab in epithelial ovarian cancer. Another clinical study (phase I/II clinical trial #NCT02758587) is to explore whether defactinib can be safely and tolerably combined with pembrolizumab (a PD-1 inhibitor) and to look for early indications of improved anticancer immunotherapy. Furthermore, clinical trial (phase II clinical trial #NCT03727880) is to investigate the effect of combination with pembrolizumab and defactinib following chemotherapy as a neoadjuvant on adjuvant treatment for resectable pancreatic ductal adenocarcinoma.

Currently, the combination of TKI and antiangiogenic agents for NSCLC patients with EGFR mutation would prolong the progression-free survival (PFS) compared to treating TKI alone group [240,241,242,243,244]. FAK takes part in angiogenesis. Therefore, the combination therapy with TKI and FAK inhibitor might be a promising strategy for non-small cell lung cancer patients harboring EGFR mutation.

## 5. Conclusion

Cancer is a complex disease that involves the simultaneous dysregulation of several biological pathways, mediated by a variety of genetic and epigenetic alterations. Targeted therapies with high efficacy and minimal side effects are necessary to treat cancers. However, tumor recurrence remains a clinical challenge over the years. The preclinical and clinical evaluations of molecularly targeted therapies against single molecule in cancer-driving pathways have demonstrated that tumors almost always exhibit inherent or acquired drug resistance, which serves as a common underlying mechanism for relapse and metastases. The cancer hallmarks summarized by Hanahan and Weinberg reveal multiple biocapacities for tumorigenesis, and all of these pathways represent therapeutic targets for cancer treatment.

FAK overexpression is a malignant feature of numerous cancer tissues and CSCs, correlated with a poor prognosis in various cancer patients. As a key coordinator of the cellular responses to environmental cues and a mitigator of cellular stresses, including those induced by therapeutic interventions, FAK is an attractive target that likely affects myriad oncogenic processes and resistance mechanisms. FAK inhibition is also likely to be more effective in the context of combination therapies, especially in tumor cells that rely on anchorage-dependent signaling initiated by the TME. Because FAK inhibits cancer activity through both kinase-dependent and -independent mechanisms, adjuvant FAK inhibitors may be able to both promote the efficacy of combination therapies while preventing the development of drug resistance. Preclinical and clinical studies remain necessary to evaluate the safety and efficacy of FAK-targeted inhibitors for cancer therapy.

## Figures and Tables

**Table 1:** Summary of preclinical studies with FAK inhibitors.

Inhibitor	Molecular Targets	Type	Cancer Types	References
BI-853520 (IN10018)	FAK	KI	Prostate cancer; breast cancer	[213,214]
GSK2256098	FAK	KI	Pancreatic cancer; ovarian cancer	[215,216]
NVP-TAC544	FAK	KI	N/A	[217]
PF-431396	FAK/PYK2	KI	Pancreatic cancer; pleural mesothelioma	[218,219]
PF-573228	FAK	KI	Pancreatic cancer; pleural mesothelioma; lung cancer	[89,219,220]
TAE226	FAK/IGF-IR	KI	Breast cancer; ovarian carcinoma; glioma	[221,222]
VS-4718 (PND-1186)	FAK/PYK2	KI	Breast cancer/ovarian cancer; pancreatic cancers	[191,223]
VS-6062 (PF-00562271)	FAK/PYK2	KI	Gliomas; pancreatic cancer; colon cancer; lung cancer; prostate cancer; breast cancer	[224,225,226]
VS-6063 (Defactinib)	FAK/PYK2	KI	Ovarian cancer	[195]
1H-Pyrrolo(2,3-b) pyridine	N/A	aKI	N/A	[227]
Compound 1 and 2	N/A	aKI	N/A	[228]
C4	FAK-VEGFR3 interaction	SI	Breast cancer	[229]
R2	FAK-p53 interaction	SI	Colorectal cancer	[230]
Y11	FAK	SI	Colon cancer and breast cancer	[231]
Y15	FAK	SI	Breast cancer; lung cancer	[232,233]

KI: kinase inhibitor; aKI: allosteric kinase inhibition; and SI: scaffold inhibitor.

**Table 2:** Summary of clinical trials with FAK inhibitors.

Drugs	Molecular Targets	Type	Cancer Types	Trial Identifiers
APG-2449	Multiple kinases	KI	Advanced Solid Cancer	NCT03917043 (I)
BI-853520 (IN10018)	FAK	KI	Metastatic Nonhematologic Malignancies	NCT01335269 (I)
Defactinib (VS-6063)	FAK/PYK2	KI	Nonhematologic Malignancies; Lung Cancer; Malignant Pleural Mesothelioma	NCT00787033 (I) NCT01943292 (I) NCT01951690 (II) NCT02004028 (II)
Defactinib (VS-6063) Avelumab	FAK/PYK2 PD-L1	KI	Epithelial Ovarian Cancer	NCT02943317 (I)
Defactinib (VS-6063) Paclitaxel	FAK/PYK2 Tubulin	KI	Ovarian Cancer	NCT01778803 (I)
Defactinib (VS-6063) Paclitaxel Carboplatin	FAK/PYK2 Tubulin DNA	KI	Ovarian Cancer	NCT03287271 (I/II)
Defactinib (VS-6063) Pembrolizumab	FAK/PYK2 PD-1	KI	Pancreatic Ductal Adenocarcinoma; Advanced Solid Malignancies	NCT02758587 (I/II) NCT03727880 (II)
Defactinib (VS-6063) Pembrolizumab Gemcitabine	FAK/PYK2 PD-1 DNA	KI	Advanced Solid Tumors; Pancreatic Cancer	NCT02546531 (I)
Defactinib (VS-6063) RO5126766	FAK/PYK2 RAF/MEK	KI	NSCLC; Solid Tumor; Low-Grade Serous Ovarian Cancer; Colorectal Cancer	NCT03875820 (I)
Defactinib (VS-6063) VS-5584	FAK/PYK2 PI3K/mTOR	KI	Relapsed Malignant Mesothelioma	NCT02372227 (I)
Defactinib (VS-6063) VS-6766	FAK/PYK2 RAF/MEK	KI	Ovarian Cancer; Metastatic Uveal Melanoma; Non-Small Cell Lung Cancer with KRAS Activating Mutation	NCT04620330 (II) NCT04625270 (II) NCT04720417 (II)
GSK2256098	FAK	KI	Solid Tumors	NCT00996671 (I) NCT01138033 (I)
GSK2256098 Trametinib	FAK MEK	KI	Advanced Solid Cancer	NCT01938443 (I)
GSK2256098 Vismodegib Capivasertib Abemaciclib	FAK Smoothened receptor AKT CDK4/CDK6	KI	Progressive Meningiomas	NCT02523014 (II)
VS-4718 (PND-1186)	FAK/PYK2	KI	Metastatic Nonhematologic Cancers; Acute Myeloid or B-Cell Acute Lymphoblastic Leukemia	NCT01849744 (I) NCT02215629 (I)
VS-4718 (PND-1186) Nab-paclitaxel Gemcitabine	FAK/PYK2 Tubulin DNA	KI	Pancreatic Cancer	NCT02651727 (I)
VS-6062 (PF-00562271)	FAK/PYK2	KI	Pancreatic, Head and Neck, Prostatic Neoplasms	NCT00666926 (I)

From www.clinicaltrials.gov, accessed on 22 December 2021. KI: kinase inhibitor.
